# Distribution of molecular strains of *Mycobacterium tuberculosis* in an intermediate burden Asia Pacific city

**DOI:** 10.1017/S0950268821001199

**Published:** 2021-05-19

**Authors:** Shui Shan Lee, Denise Pui Chung Chan, Ngai Sze Wong, Grace Chung Yan Lui, Kin Wang To, Joseph Kai Man Kam

**Affiliations:** 1 Stanley Ho Centre for Emerging Infectious Diseases, The Chinese University of Hong Kong, Hong Kong, China; 2 Department of Medicine and Therapeutics, The Chinese University of Hong Kong, Hong Kong, China

**Keywords:** Infectious disease control, molecular epidemiology, respiratory infections, tuberculosis (TB)

## Abstract

Hong Kong is an intermediate tuberculosis (TB) burden city in Asia Pacific with slow decline of case notification in the last decade. By 24-loci mycobacterial interspersed repetitive units – variable number of tandem repeats genotyping, we examined 534 *Mycobacterium tuberculosis* isolates collected from culture-positive hospitalised TB patients in a 1.7 million population geographic region in the city. Overall, 286 (75%) were classified as Beijing genotype, of which 216 (76%) and 59 (21%) belonged to modern and ancient sub-lineage, respectively. Only two cases were genetically clustered while spatial clustering was absent. Male gender, permanent residency in Hong Kong and born in Hong Kong or Mainland China were associated with Beijing genotype. The high prevalence of Beijing modern lineage was similar to that in East Asia, which reflected the pattern resulting from population migration. The paucity of clustering suggested that reactivation accounted for most of the TB disease cases, which was and echoed by observation that half were 60 years old or above, and the presence of co-morbid medical conditions. The predominance of reactivation TB cases in intermediate burden localities implies that the detection and control of latent TB infection would be the major challenge in achieving TB elimination.

## Short report

Molecular epidemiologic analyses are particularly useful in tracking *Mycobacterium tuberculosis* (MTB) spread in high burden areas and for investigating its emergence in low prevalence settings. Hong Kong is a city with intermediate tuberculosis (TB) burden in the Asia Pacific region [1]. In the past decade the TB notification rate in Hong Kong averaged between 55 and 65 per 100 000 population, with a consistently higher rate of over 90 per 100 000 in elderly aged 65 or above (https://www.chp.gov.hk/en/statistics/submenu/). The notable slow decline in TB notification could be attributed to the high prevalence of MTB infection in older individuals and a 17-fold higher risk of reactivation compared to western countries like the United Kingdom [2]. Compared to high and low burden settings, the molecular epidemiology of TB in settings predominated by reactivation diseases has so far received little attention. We conducted a molecular study to characterise the epidemiologic pattern of TB in this intermediate TB burden city.

Between April 2015 and September 2016, we collected MTB strains isolated from culture-positive TB patients hospitalised in the geographic region of New Territories East, home to 1.7 million population in Hong Kong. The MTB lineages were determined by 24-loci Mycobacterial interspersed repetitive units – variable number of tandem repeats (MIRU-VNTR) genotyping. The corresponding number of repetitions of MIRU-VNTR loci of each strain was analysed by MIRU-VNTRplus (https://www.miru-vntrplus.org/MIRU/index.faces). A minimum spanning tree was constructed based on the 24-loci MIRU-VNTR genotyping data. Isolates with identical MIRU-VNTR genotypes were defined as belonging to the same cluster. Location data of patients were geocoded and aggregated in geographic units in ArcGIS, followed by the determination of Pearson's correlation with the distribution of the general male population. Patient characteristics between MIRU-VNTR genotypes (Beijing and non-Beijing), and between Beijing sub-lineages (modern and ancient) were compared in bivariable logistic regression and multivariable logistic regression models with gender as a confounder in SPSS. Ethical approval has been obtained from the Joint Chinese University of Hong Kong – New Territories East Cluster Clinical Research Ethics Committee (CRE2014.572).

Out of a total of 534 MTB isolates collected, unique isolates from 382 patients were included in the final analysis. During the study period, about 4000 TB cases were notified in the whole territory of Hong Kong per year. The sample size accounted for an estimated 60% of all culture-positive cases detected in the geographical region in the same period. The male-to-female ratio was 2.2, half of which aged ≥60 years. Other socio-demographic characteristics were: born locally (*n* = 41, 53%), permanent residence in Hong Kong (*n* = 52, 75%), living in long-term care facilities (LTCF) (*n* = 24, 6%). There was no spatial clustering of TB cases by residence locations. Smoking and alcohol habits were reported by 25% and 21%, respectively. Clinically, 269 (70%) presented with pulmonary diseases. Concurrent medical conditions were diagnosed in 190 (50%). Only 2 (1%) were HIV infected. There were no differences in the proportional distribution of underlying medical conditions between MIRU-VNTR genotypes.

**Fig. 1.**
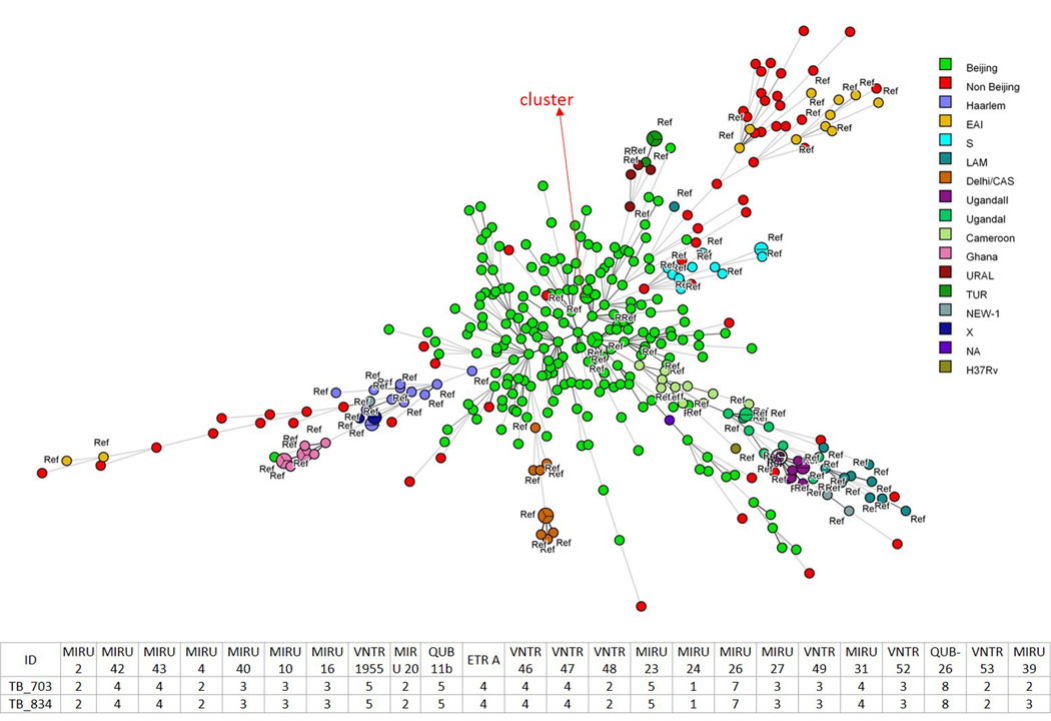
Minimum spanning tree based on the 24-MIRU-VNTR genotyping data of 382 MTB isolates from Hong Kong. The red circle denotes the non-Beijing family strain, while the green circle denotes the Beijing family strain.

Overall, 286 (75%) were classified as Beijing genotype, of which 216 (76%) and 59 (21%) belonged to modern and ancient sub-lineage, respectively. The median age of patients with Beijing and non-Beijing genotype was similar (60 *vs* 56.5, *P* = 0.15). Only two cases were clustered (Fig. 1) by MIRU-VNTR. Both were male of age 24 and 56, non-smokers, without anti-mycobacterial resistance. The 24-loci MIRU-VNTR system gave a high discriminatory power for the loci with an overall Hunter−Gaston Discriminatory Index (HGDI) of 0.9594 [3]. There was positive but marginally significant correlation between the proportion of Beijing genotype isolates and that of male general population in corresponding geographic units (*r* = 0.23, *P* = 0.058). Male gender, permanent residency in Hong Kong, and born in Hong Kong or Mainland China were associated with Beijing genotype (Table 1). There were no socio-demographic differences between modern and ancient Beijing sub-lineage. Twenty-eight strains (7%) were resistant to one of the four first-line anti-mycobacterial compounds, while 14 (4%) were resistant to more than one compound. Non-Beijing genotype gave a slightly higher prevalence of isoniazid resistance (8% *vs* 3%, adjusted OR (aOR) with gender as a confounder = 0.34, 95% CI 0.13–0.92, *P* = 0.034) (Table 1). Multidrug-resistant TB was similarly uncommon in non-Beijing (2/95, 2%) and Beijing genotype (2/286, 1%) (aOR 0.36, 95% CI 0.05–2.67). There was no difference in the resistance pattern between modern and ancient sub-lineage.

**Table 1.** Comparison between Beijing and non-Beijing genotype *M. tuberculosis* cases admitted to a major regional hospital in Hong Kong, 2015–2016 (*n* = 382)

	Non-Beijing (*n* = 96)		Beijing (*n* = 286)		Odds ratio (OR)		Adjusted OR (aOR)^a^	
	*n*	%	n	%	OR	(95% CI)	aOR	(95% CI)
Gender	*n* = 96		*n* = 286					
Female	40	42%	79	28%	*ref*			
Male	56	58%	207	72%	1.87*	(1.16–3.03)	/	
Age group (year-old)	*n* = 96		*n* = 286					
<35	18	19%	41	14%	*ref*		*ref*	
35–49	13	14%	30	10%	1.01	(0.43–2.38)	1.01	(0.42–2.38)
50–59	25	26%	63	22%	1.11	(0.54–2.28)	0.93	(0.44–1.96)
60–69	20	21%	62	22%	1.36	(0.64–2.88)	1.15	(0.53–2.48)
70–79	10	10%	46	16%	2.02	(0.84–4.87)	1.65	(0.67–4.07)
≥80	10	10%	44	15%	1.93	(0.80–4.67)	1.70	(0.70–4.16)
Admission from LTCF	*n* = 96		*n* = 286					
No	89	93%	269	94%	*ref*		*ref*	
Yes	7	7%	17	6%	0.80	(0.32–2.00)	0.77	(0.30–1.92)
Birth place	*n* = 30		*n* = 48					
Hong Kong and China	20	67%	43	90%	*ref*		*ref*	
Others	10	33%	5	10%	0.23*	(0.07–0.77)	0.36	(0.10–1.28)
Non-permanent residents	*n* = 23		*n* = 46					
No	13	57%	39	85%	*ref*		*ref*	
Yes	10	43%	7	15%	0.23*	(0.07–0.74)	0.27*	(0.08–0.94)
Smoking	*n* = 93		*n* = 265					
Non-smoker	49	53%	119	45%	*ref*		*ref*	
Ex-smoker	25	27%	74	28%	1.22	(0.69–2.14)	0.78	(0.40–1.54)
Current smoker	19	20%	72	27%	1.56	(0.85–2.86)	1.05	(0.52–2.10)
Alcohol	*n* = 85		*n* = 246					
Non-drinker	55	65%	163	66%	*ref*		*ref*	
Ex-drinker	12	14%	31	13%	0.87	(0.42–1.81)	0.54	(0.24–1.21)
Current drinker	18	21%	52	21%	0.97	(0.53–1.81)	0.63	(0.32–1.26)
Co-morbidities	*n* = 96		*n* = 286					
Cardiovascular diseases	12	13%	50^b^	18%	1.49	(0.76–2.93)	1.31	(0.66–2.62)
Cancer	18	19%	36	13%	0.62	(0.34–1.16)	0.55	(0.29–1.04)
Chronic respiratory diseases	9	9%	33	12%	1.26	(0.58–2.74)	1.09	(0.50–2.41)
Diabetes	20	21%	82	29%	1.53	(0.88–2.66)	1.43	(0.81–2.50)
HIV infection	0^c^	0%	2^d^	1%	/		/	
Type of TB	*n* = 96		*n* = 286					
Pulmonary	67	70%	202	71%	*ref*		*ref*	
Extrapulmonary	28	29%	75	26%	0.89	(0.53–1.49)	1.01	(0.60–1.72)
Both	1	1%	9	3%	2.99	(0.37–24.00)	3.12	(0.38–25.32)
TB drug resistance	*n* = 95		*n* = 286					
Isoniazid	8	8%	9	3%	0.35*	(0.13–0.94)	0.34*	(0.13–0.92)
Streptomycin	7	7%	29	10%	1.42	(0.6–3.35)	1.36	(0.57–3.24)
Rifampicin	2	2%	3	1%	0.49	(0.08–3.00)	0.52	(0.08–3.20)
Ethambutol	0	0%	2	1%	/			
TB drug resistance (any)	*n* = 95		*n* = 286					
No	86	91%	253	88%	*ref*		*ref*	
Yes	9	9%	33	12%	1.25	(0.57–2.71)	1.18	(0.54–2.59)
Multidrug resistance^e^								
No	93	98%	284	99%	*ref*		*ref*	
Yes	2	2%	2	1%	0.33	0.05–2.36	0.36	(0.05–2.67)

* *P* < 0.05.

a Adjusted by gender in multivariable logistic regression.

b 1 missing.

c 37 missing.

d 92 missing.

e Multidrug resistance refers to Isoniazid and Rifampicin resistance with/without other regimen resistance.

In our cohort, Beijing genotype predominated the prevalence of which matching that in other Asian countries [4]. The association between Beijing genotype and the birthplace of Hong Kong / Mainland China lends support to the distribution of MTB that is geographically structured according to the history of migration. Less than 1% had HIV co-infection, corresponding to the limited dissemination of HIV in Hong Kong. A majority (75.8%) of the Beijing genotype cases belonged to modern lineage, similar to that in parts of Japan in East Asia [5], but higher than South East Asian countries like Vietnam [6]. The discrepancy could again be a result of population migration in the recent decades, while the geographic origin of modern lineage remains speculative [7]. The delineation of modern Beijing lineage signalled the emergence of virulent MTB which may be more fit for further transmission [8].

One unique finding of the analyses was the paucity of detectable molecular or spatial clusters. Compared to a similar study reported about 20 years ago in Hong Kong [9], clustering has decreased from over 10% to below 1%. The lack of clustering in our cohort suggested that reactivation could have accounted for an increasing proportion of TB disease cases. This is supported also by the observation that half of the TB patients were of older age at 60 or above, and the presence of co-morbid medical conditions. With a higher discriminatory power, whole genome sequencing (WGS) could be better able to detect transmission events but its role in refining the investigation of reactivation TB is unknown [10, 11]. The control of TB reactivation at population level is a challenge. From our findings, non-clustering MTB isolates could be treated as a surrogate for estimating the prevalence of reactivation diseases among all active TB cases. It would be necessary to develop intervention comprising LTBI screening that targets elderly people, immunocompromised patients and those with comorbid conditions. The predominance of reactivation of TB cases in intermediate burden localities implies that strategic development of interventions for controlling LTBI would be crucial in advocating TB elimination [12].

## Data Availability

The data that support the findings of this study are available from the authors with the permission of Ethics Committee.
